# Osseous metaplasia in an ulcerating tubular adenoma of the colon: a case report

**DOI:** 10.1186/1752-1947-2-130

**Published:** 2008-04-29

**Authors:** Victoria White, Aidan G Shaw, Gillian M Tierney, Jonathan N Lund, David Semeraro

**Affiliations:** 1 Department of Colorectal Surgery, Derby City General Hospital, Derby, UK; 2 School of Graduate Medicine and Health, University of Nottingham United Kingdom, Derby, DE22 3DT, UK; 3 Department of Histopathology, Derby City General Hospital, Derby, UK

## Abstract

**Introduction:**

Heterotopic bone is rarely found in the gastrointestinal tract. Here we report a rare case of metaplastic ossification within a benign ulcerating adenoma and review the literature concerning the aetiology.

**Case presentation:**

A 63-year-old woman, who presented with a history of melaena, was found at colonoscopy to have a pedunculated ulcerating polyp. Histological examination demonstrated multiple areas of osseous metaplasia within the polyp stroma.

**Conclusion:**

Heterotopic ossification in colonic adenomas is a particularly rare phenomenon, with the majority of cases occurring within malignant lesions. The suggested mechanisms for its aetiology still remain unclear.

## Introduction

Heterotopic bone is rarely found in the gastrointestinal tract. The majority of reported cases are associated with malignant lesions [1-6]. There are few reports of osseous metaplasia in benign colonic polyps [7-12]. Various mechanisms have been proposed on the aetiology yet it still remains poorly understood. Here we report a case of osseous metaplasia in a benign ulcerating adenoma and a review of the literature on suggested mechanisms for its aetiology.

## Case presentation

A 63-year-old woman presented to her general practitioner with a history of intermittent melaena. She was drinking two to three litres of gin per week and was taking ibuprofen for cervical spondylosis. She had a past medical history of alcoholic liver disease and, 6 years previously, a gastroscopy had revealed oesphagitis and duodenitis.

A repeat gastroscopy revealed no abnormality. A colonoscopy was performed which revealed a pedunculated polyp in the proximal transverse colon which was subsequently excised, retrieved and sent for histological examination. The patient has subsequently had no further admissions to hospital or episodes of melaena.

**Figure 1:**
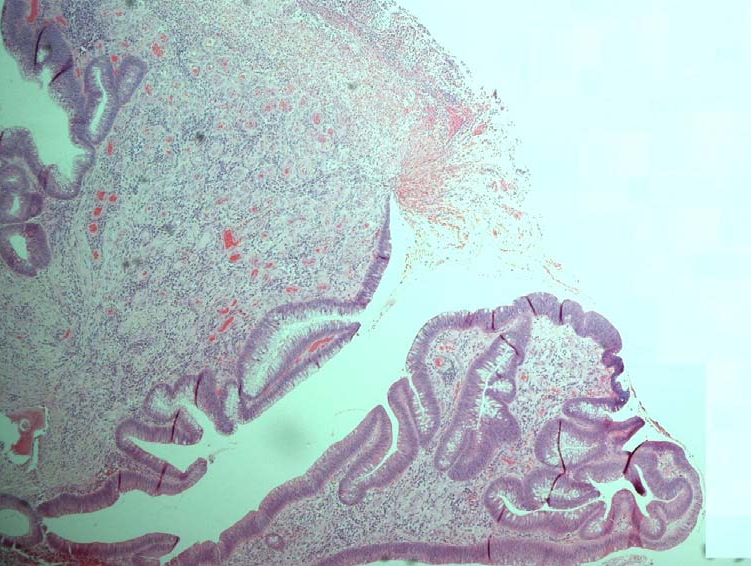
Histopathological specimen of the polyp demonstrating adenomatous surface epithelium and slough and inflammatory debris in an area of surface ulceration.

**Figure 2:**
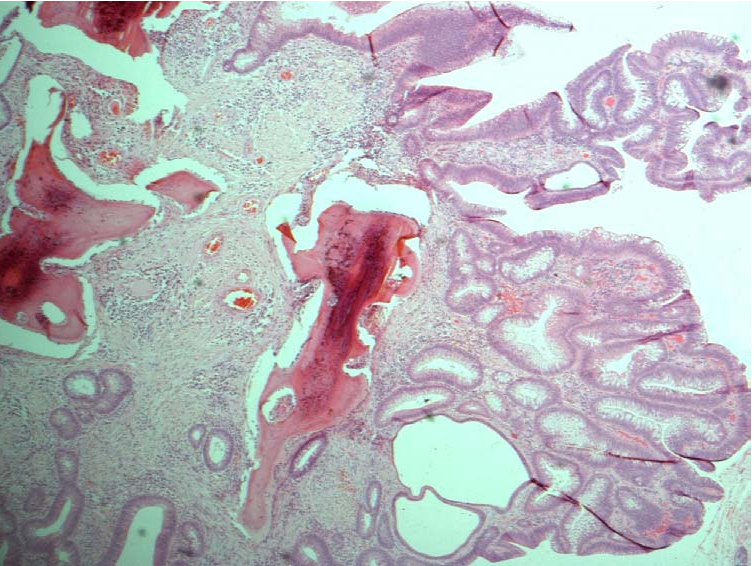
Histopathological specimen of the polyp demonstrating adenomatous surface epithelium and osseous metaplasia with surface osteoclasts.

All sections of the specimen demonstrated an adenomatous polyp with a mostly tubular growth pattern and moderate epithelial dysplasia. Areas of surface ulceration with granulation tissue and slough were also noted (Figure 1). The polyp stroma contained multiple areas of osseous metaplasia; the polyp base demonstrated normal mucosa with complete excision margins (Figure 2).

## Discussion

Osseous metaplasia occurs outside the skeletal system in fibrodysplasia ossificans [13]. The incidental finding of bone in histological specimens, although extremely interesting, seems to have no clinical significance [8,10]. Heterotopic ossification in colonic adenomas is a particularly rare phenomenon. There are only three other cases reported of ossification specifically in a tubular adenomas [7,8,12]. Osseous metaplasia can also occur in juvenile polyps [8], Peutz-Jeghers Syndrome [11] and inflammatory polyps [9] as well as in malignant lesions [1-6].

How this ectopic ossification occurs is unknown. It may be that the osteoblasts needed to lay down bone differentiate from fibroblasts or other precursor cells. Local osteogenic factors then stimulate these osteoblasts to incorporate collagen fibres already found at the site into new bone. Bone remodelling dependent on the balance between osteoblasts and osteoclasts occurs in areas of ectopic bone as it does in the normal skeleton [9].

The adenomatous polyp removed from our patient had surface ulceration alongside areas of granulation and slough. It may be that the osteogenic stimulation was a result of the inflammatory process. Inflammation has previously been suggested as a trigger in a case of osseous metaplasia in an ulcer in Barrett's oesophagus [10] and in a rectal polyp found to have inflammatory infiltrate alongside the foci of osseous metaplasia [9]. Apostolidis et al. report two theories as to the cause of heterotopic bone found in abdominal incision scars [14]. The first, a suggestion that particles of bone are inoculated into the wound during surgery from the xiphoid process or symphysis pubis, is hard to relate to our case. The second theory, suggesting that osseous metaplasia is the result of differentiation of immature connective tissue to osteoblasts is perhaps more applicable. This differentiation, as Apostolidis et al. suggest, may be a reaction to local injury [14]. The areas of ulceration and granulation tissue found in the polyp from our patient may be as a result of local damage. Osseous metaplasia may have then occurred secondary to this damage. In a case of osseous metaplasia in a benign ovarian cyst the authors also suggest that the bone formation can be due to a reaction to tissue damage and repair [15]. It has also been reported that substances released by abnormal epithelial cells can go on to induce ossification [8]. However, these substances remain to be identified.

## Conclusion

Heterotopic ossification in colonic adenomas is a particularly rare phenomenon, with the majority of cases occurring within malignant lesions. The suggested mechanisms for its aetiology still remain unclear.

## Competing interests

The authors declare that they have no competing interests.

## Authors' contributions

VW and AS wrote the manuscript. GT performed the colonscopy. AS, JL, GT and DS reviewed the literature. All authors contributed intellectual content and have read and approved the final manuscript.

## Consent

Written informed consent was obtained from the patient for publication of this case report and accompanying images. A copy of the written consent is available for review by the Editor-in-Chief of this journal.
